# Prognostic Significance and Treatment Implications of Minimal Residual Disease Studies in Philadelphia-Negative Adult Acute Lymphoblastic Leukemia

**DOI:** 10.4084/MJHID.2014.062

**Published:** 2014-09-01

**Authors:** Orietta Spinelli, Manuela Tosi, Barbara Peruta, Marie Lorena Guinea Montalvo, Elena Maino, Anna Maria Scattolin, Margherita Parolini, Piera Viero, Alessandro Rambaldi, Renato Bassan

**Affiliations:** 1 Hematology and Bone Marrow Transplant Unit of Azienda Ospedaliera Papa Giovanni XXIII, Bergamo, Italy; 2 Hematology and Bone Marrow Transplant Unit, Ospedale dell’Angelo e SS. Giovanni e Paolo, Mestre-Venezia, Italy

## Abstract

Acute lymphoblastic leukemia (ALL) is curable in about 40–50% of adult patients, however this is subject to ample variations owing to several host- and disease-related prognostic characteristics. Currently, the study of minimal residual disease (MRD) following induction and early consolidation therapy stands out as the most sensitive individual prognostic marker to define the risk of relapse following the achievement of remission, and ultimately that of treatment failure or success. Because substantial therapeutic advancement is now being achieved using intensified pediatric-type regimens, MRD analysis is especially useful to orientate stem cell transplantation choices. These strategic innovations are progressively leading to greater than 50% cure rates.

## Introduction

Philadelphia-negative (Ph-) ALL in adults is a relatively rare neoplasm with an overall survival rate of 40% or slightly higher in adult patients with an age range between 15 to 60 years. Obtaining an early complete remission (CR) and avoiding relapse are the two essential therapeutic steps to achieve cure. Although the vast majority of patients will achieve CR, nearly half of them are at risk of relapse in relation with the individual risk profile.^1^ The several risk factors considered by leading European Groups are shown in Table 1. Once CR is achieved, the risk-adapted approach is dedicated to identify the patients who are (more) likely to benefit from conventional chemotherapy, that carries the lowest risk of treatment-related morbidity and mortality, and those for whom allogeneic stem cell transplantation (allo-SCT) is indicated, despite the higher toxicity associated with this procedure (at least 10–15% transplant-related mortality at 1–3 years, and up to 30% in selected bad-risk subsets).^2^ The paradigm of therapy optimization is expected to be further improved as much as our understanding of the mechanisms underlying resistance and relapse is increased, and novel therapeutics for specific risk and disease subsets are developed.

## ALL Risk Subsets

Ph- ALL is a disease of B- or T-cell precursors and is mostly defined by a rapid immunophenotypic analysis of blast cell populations. B-lineage ALL (80%) usually expresses cytoplasmic CD22, CD19, CD10 in the “common” subset, and clonal surface immunoglobulin in Burkitt leukemia; T-lineage ALL (20%) does express cytoplasmic CD3, CD7, CD1a+ in cortical T-ALL, and surface CD3 in mature T-ALL. Cytogenetics and genetics are necessary to distinguish between Philadelphia chromosome/BCR-ABL1 positive (Ph+) and Ph- ALL, or identify other high-risk abnormalities such as t(4;11)/KMT2A rearrangements, monosomy 7, hypodiploidy and IKZF1 gene deletion. Recognizing Ph+ ALL and Burkitt leukemia is fundamental, because these formerly high-risk subsets require and can greatly benefit from different, highly specific treatments. ALL subsets are variously considered in the risk sub-classification of different study Groups (Table 1).

## Treatment Steps and the Role of MRD Analysis

**Figure 1:**
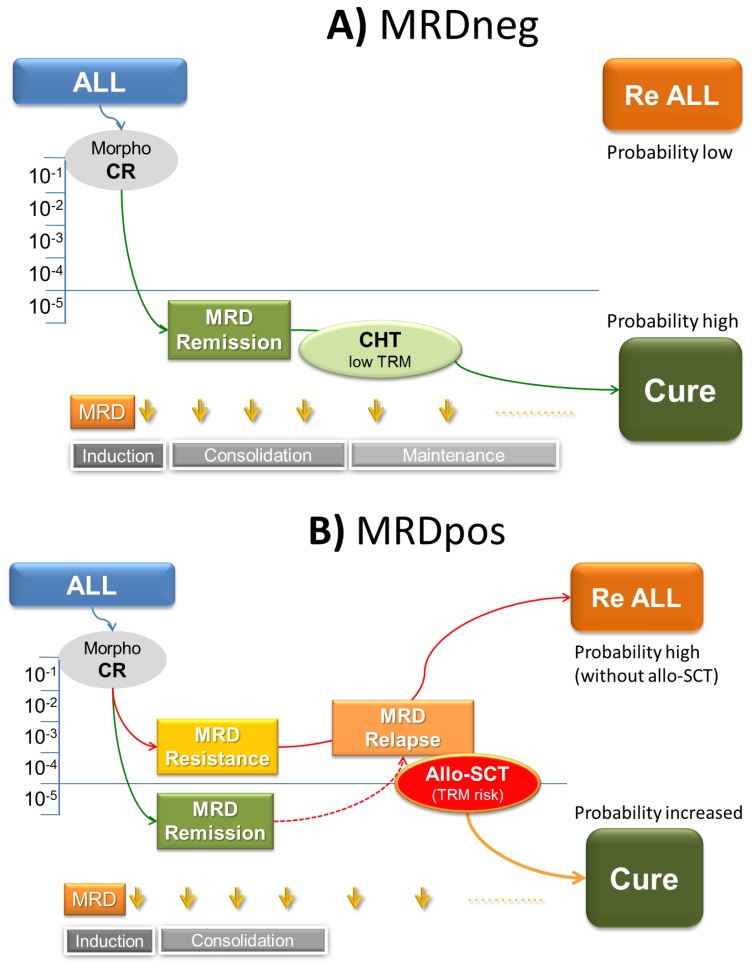
The relationship between MRD-based risk definition and treatment in adult ALL. A) CR patients achieving good/complete and durable MRD response are at low risk of recurrence and can achieve cure on standard chemotherapy only. B) CR patients with insufficient MRD clearing or MRD relapse subsequent to MRD remission, are at high risk of relapse and cannot be cured by chemotherapy. A significant proportion of these cases can be effectively rescued by an allo-SCT.

The disease response to chemotherapy remains the primary determinant of outcome. It would be possible as demonstrated by several recent trials, to lower the rate of refractory/relapsed (R/R) ALL and improve overall treatment results by moving from standard adult-type programs to pediatric-derived therapy (PDT), as extensively reviewed by J Ribera in this issue of the Journal. Apart from that, the response kinetics to the early components of chemotherapy can be assessed through MRD analysis at predefined treatment steps, using either flow cytometry that identifies leukemia-associated immunophenotype or RQ-PCR methodology that detects abnormal fusion genes or case-specific gene rearrangements. Post- induction MRD is the most important prognostic indicator, basically superseding any other pretreatment risk factor for relapse.^3^ For this reason, a prospective MRD analysis can be used to enable recognition of “true” high-risk (HR) patients with suboptimal MRD response, to whom offer allo-SCT.^4^,^5^ On the other hand patients showing complete MRD response following induction/consolidation chemotherapy, at “true” standard-risk (SR), can avoid allo-SCT (simultaneously lowering treatment mortality).^4^ The general outline of this strategy is highlighted in Figure 1. In this regard terms such as molecular complete remission (molCR) and resistance (or relapse) indicate absent or low MRD signals (<10^−4^) versus persistence or rise of MRD above this critical threshold.^5^ The relationship between MRD positivity and relapse is a strict one, a full blown relapse occurring within few weeks to months from MRD reappearance despite intensive treatment.^6^

## MRD Detection: Molecular Analysis of Gene Rearrangements

MRD evaluation can be performed in several ways with different limits of detection and standardization (Table 2). The most commonly used technique is the molecular study based of antigen-receptor gene rearrangements. During B and T cell development immunoglobulin (Ig) and T-cell receptor (TCR) gene segments (V, D and J) undergo multiple gene rearrangements eventually generating functional receptors. In this process some nucleotides are randomly deleted or inserted at junctional sites of each segment, leading to final receptor sequences unique to each B or T lymphocyte. In case of neoplastic evolution of a single lymphoid cell, all the derived progeny will express the same receptor sequence (clonality). Although Ig rearrangements are mostly found in B-cells and TCR rearrangements in T-lymphocytes, cross-lineage antigen receptor rearrangements are frequent. Up to 90% of precursor B-ALL may express TCR gene rearrangements,^7^ whereas a lower proportion of T-ALL (20%) shows Ig rearrangements.^8^ To identify these highly sensitive and case-specific molecular sequences at diagnosis, genomic DNA derived from leukemic cells undergoes PCR amplification with consensus oligonucleotides which recognize different V, D and J family fragments of antigen receptors. The identified Ig and TCR rearrangements are then analyzed by heteroduplex or gene scan^9^ to assess clonality. Clonal PCR fragments are then sequenced by Sanger method. Junctional regions defined by sequencing are used to design clone-specific oligonucleotide for MRD monitoring mostly by Real-time quantitative PCR (RQ-PCR). Amplification conditions and test sensitivity for each clone-specific oligonucleotide are established on diagnostic material serially diluted in normal mononuclear cells. The sensitivity of the test can reach 10^−5^, i.e. one leukemic cell can be detected out of 100,000 normal cells, and therefore, leukemia cell dilutions are used to quantify MRD in bone marrow samples collected during treatment. This technology can generate at least a single sensitive molecular probe (i.e., 10^−4^, detecting one leukemic cell out of 10,000 normal cells) suitable for MRD analysis in up to 90% of pediatric^10^ and adult^4^,^11^,^12^ ALL patients. This technology was extensively standardized during the last 15 years within the EuroMRD group (previously known as ESG-MRD-ALL study group), that established optimal technical conditions and interpretation guidelines^9^ to favor an easier and homogeneous application of MRD studies within different treatment protocols. In addition to the high sensitivity and standardization level achieved, another advantage of the molecular technique is that being based on DNA analysis it allows the necessary shipping time to centralize samples at qualified laboratories for multicenter studies.

## MRD Detection: Molecular Analysis of Gene Fusion Transcripts

Another method for molecular MRD detection and monitoring is based on fusion transcript analysis. Forty percent of ALL samples bear chromosomal translocations generating chimeric transcripts that can be used to discriminate leukemic cells from normal cells. The most common translocation product found in adult ALL is that of Ph+ ALL, i.e. the BCR-ABL1 transcript (25–30%), whereas the most common chimeric transcript in pediatric patients is represented by ETV6-RUNX1 that accounts for 25–30% of translocated childhood ALL. Other fusion transcripts are KMT2A-AFF1 and TCF3-PBX1 each accounting from 3–8% of adult and pediatric ALL.^13^ Due to the large DNA portion in which translocation breakpoints occur, patient specific tool for MRD evaluation cannot be easily obtained. Interestingly, the RNA splicing process produces in all the patients the same fusion transcript or few splicing variants. This offer the opportunity to apply the same primer set to all the patients bearing the same translocation leading to an easy and rapid fusion transcript evaluation at diagnosis and during treatment.^14^ Another advantage of this approach is represented by the stable association between the gene fusion and the leukemic clone because of its involvement in neoplastic transformation.

## Problems and Pitfalls of Molecular MRD Analysis

The generation of clone-specific Ig/TCR probes for MRD evaluation is quite expensive, time-consuming and requires experienced personnel. Therefore, this method can be successfully applied only by specialized laboratories processing several cases per year. Other pitfalls have to be considered: in lymphoid cells receptor-antigen rearrangements are not linked to the oncogenic process itself and can persist during the treatment of ALL, possibly leading to falsely positive MRD results.^15^ The real occurrence of this phenomenon is unknown. Furthermore, leukemic sub-clones with a different rearrangement pattern already present at low, undetectable levels at diagnosis can emerge during treatment.^16^,^17^ In addition, false positive results can be obtained when massive, post-chemotherapy lymphocyte regeneration is present.^18^–^20^ Also the analysis of abnormal fusion gene transcripts presents some limitations. First, the utilization of RNA as RQ-PCR target that is more prone to degradation, impairing in some cases a correct MRD detection. Another risk is the cross-contamination during the RQ-PCR procedure possibly leading to false positive results. Furthermore, the amount of transcript is not directly related to leukemic cell number but rather depends on the transcription level that can differ among cases, rendering the MRD measurement less accurate and less comparable between patients.^14^ Cooperative efforts are ongoing to optimize BCR-ABL1 detection and to harmonize MRD expression by the EWALL and ESG-MRD-ALL (now fused into the EuroMRD study group).^21^

## MRD Detection: Flow Cytometry Analysis

A further MRD detection method is represented by multi-parametric flow cytometry (MFC). This approach takes advantage from the presence of proteic epitopes on the cell surface, that are differently expressed by B- and T-lymphoblasts and are sequentially acquired during cell development. The study of these molecules with specific diagnostic antibodies can identify different stages of development of normal lymphocytes as well as leukemic cells in which an aberrant or asynchronous expression can be found. This leukemia-associated immunophenotype (LAIP) has to be identified at diagnosis before any therapy in each ALL case, by comparing the marker profile of leukemia cells to reference bone marrow samples. This approach is successful in a vast proportion of cases (>90%) and can reach a sensitivity of 10^−3–^10^−4^ (one leukemic cell out of 1000–10,000 normal cells).^22^–^24^ The MFC analysis is quick, can release MRD evaluations suitable for clinical decisions in few hours, and is, therefore, particularly useful to assess the therapeutic response following the first two induction weeks.^25^

## Problems and Pitfalls of MFC MRD Analysis

Despite its rapidity and high applicability rate, MFC also has some limitations. It requires fresh, viable cells, which could be a problem within multicenter studies in which samples are sent to a reference laboratory, as shipping can take more than 24 hours. Furthermore, the sensitivity is greatly dependent on the number of evaluated cells (i.e. number of acquired events), while specificity depends on the presence of true leukemic cells in the MRD samples. In post-induction, a regenerating bone marrow can contain a large number of immature normal lymphoid cells leading to false positive results.^18^–^20^ Another phenomenon that can impair MRD detection by MFC is a phenotypic shift occurring with chemotherapy and modifying the leukemic antigen profile.^26^ The latter problem can be partially overcome by using multiple sets of markers for each case. MFC-based MRD studies underwent a large effort to standardization of methodology during the last decade, again within the EuroFlow Consortium.^27^,^28^ Nevertheless, a reliable MFC MRD evaluation can only be performed in a specialized laboratory run by highly experienced operators.

## MRD Detection: the Novelty of Next Generation Sequencing

The recent availability of high-throughput next generation sequencing (NGS) provides an opportunity to explore new methods for MRD detection and monitoring. Similar to the conventional molecular techniques, the first NGS studies were based on Ig/TCR gene rearrangements amplification by multiple sets of oligonucleotides. The difference is that amplicons are sequenced by NGS instead of Sanger sequencing, thus giving the opportunity to identify dominant clones, as well as minor clones. Follow up samples are then studied with the same amplification and sequencing approach without the need of performing a patient, clone–specific RQ-PCR assay. To calculate the absolute number of rearranged molecules, it is necessary to add specific reference sequences.^29^–^32^ NGS based methods are potentially more accurate and sensitive than RQ-PCR based technology and can increase the accuracy of MRD analysis for clinical purposes, but some aspects remain to be specifically addressed. These refer to quantity and type of diagnostic material, internal controls, primer design and combination for multiplex reactions, background definition, maximal and reproducible sensitivity determination, sequence quality parameters, error correction and bioinformatics data analysis. An European Consortium, named EuroClonality-NGS Consortium, has been recently created to address all these aspects in a scientifically independent way. The consortium consists of laboratories already experienced in designing and testing assays for Ig/TCR rearrangements detection and their evaluation within clinical trials.^33^

## Clinical and Therapeutic Implications of MRD Analysis

Since MRD analysis is adopted to refine the individual prognostic profile (Table 1), it is expected that a significant proportion of SR patients, without any known traditional risk factor, will be found MRD positive (MRDpos) at convenient, prefixed MRD study time-points. These patients, still harboring many ALL cells in remission marrows, usually more than 10^−4^ and up to 10^−2^, will experience disease relapse in an usually short lapse of time, despite continuation of intensive therapy (Figure 1). This is the case where MRD analysis can explain why up to 40% of clinically SR patients do eventually relapse. Because persistent MRD reflects chemo-resistance, there is little point in continuing the same therapy, given the increasing risk of recurrence over time. On the contrary there are patients with HR ALL, who exhibit prompt and complete MRD response, a finding that in all MRD-based trials is associated with a high probability of cure. Therefore, an allo-SCT may be therapeutically redundant in MRD negative (MRDneg) patients while it must be seriously considered in MRDpos ones, in order to overcome the poor outlook associated with the persistence and rise of residual disease. Terms such as molecular CR (molCR) and molecular failure (molFail) were coined to distinguish these opposite conditions.^5^ In essence the MRD analysis refines our ability to recognize which patients can be rather safely excluded from allo-SCT, because obtaining a satisfactory cure rate with chemotherapy only and without the risk of SCT-related mortality. Instead, MRDpos patients would partially benefit from an allo-SCT. Against this background, we review the evidence gathered by prospective, MRD-based clinical trials in adult Ph- ALL.

## Clinical Application of MRD Study in Ph- Adult ALL: Prospective Clinical Trials

The published data concern 580 patients from GMALL trials,^5^ 136 patients from a NILG trial,^4^,^34^ and 161 patients from a PETHEMA trial,^35^ for a total of 877 adult patients with Ph- ALL valuable for MRD response to induction/consolidation therapy and eligible to an MRD-driven treatment strategy with regard to the final decision between allo-SCT or standard chemotherapy in CR1. Notably, these patients are a variable proportion of all patients in each study, the reasons for exclusion from the MRD study being reported in the appropriate reference and ranging from lack of suitable MRD markers to early treatment failure, etc. While GMALL and NILG trials adopted the molecular MRD analysis, the PETHEMA trial adopted the MFC analysis. In addition, in a GRAALL trial the molecular MRD response was prospectively assessed in a further 423 patients, although this information was not used to allocate them to different treatments.^36^ Nevertheless selected results from this study will be reviewed because highly relevant to this discussion. The main results from the trials quoted are shown in Table 3. As shown, the GMALL, NILG and PETHEMA studies had a different design about patient selection, methods and timing of MRD analysis, and therapeutic decisions based on MRD analysis results. In particular an MRD- based treatment was planned for SR subsets only in the GMALL trial, all risk subsets except t(4;11) positive ALL in the NILG trial, and HR subsets only in the PETHEMA trial. Apart from that and the different treatment protocols, MRD was confirmed in all studies as the most significant risk factor for relapse, with a significantly better outcome for MRDneg patients, over the MRDpos group, regardless of allo-SCT being performed or not. Interestingly, the GRAALL study confirmed an interaction between MRD and oncogene expression, the risk of relapse being the highest for MRDpos patients with KMT2A positivity or IKZF1-deleted B-ALL, or with NOTCH1/FBW7^WT^ and/or N/K-RAS-mutated and/or PTEN-altered T-ALL.^36^ Again, a subset analysis of the NILG trial restricted to patients with CD20+ B-ALL demonstrated that patients with this poor-risk ALL subset have an excellent outcome even without allo-SCT when MRD negativity is achieved.^37^

## Effects of allo-SCT in MRDpos Subsets

As a rule MRDpos patients rely on allo-SCT for survival and will relapse rapidly unless transplantation or other alternative treatments are successfully applied. In order to do so, it is mandatory to search for an HLA-compatible related or unrelated donor (or another source: cord blood) as soon as possible, to have the shortest possible interval between detection of MRD positivity and allotransplantation. To save time, because the CR rate is about 90% in adult ALL, the donor search should initiate at diagnosis in all patients, without waiting for CR. Then, the conditioning regimen, whenever possible, should be intensified using either total body irradiation (TBI) ≥13 Gy or etoposide, which in a large retrospective analysis of allotransplantation in advanced-stage ALL (of which MRDpos ALL may represent a preclinical variant) were more effective than TBI <13 Gy (with cyclophosphamide) or cyclophosphamide (with TBI ≥13 Gy), respectively.^38^ Subsequently, it is wise to recheck MRD rapidly post-transplantation, to decide about tapering of immune suppression and/or start of donor lymphocyte infusions. The results of the three prospective European trials, albeit with some differences, confirmed the partial success of allo-SCT in MRDpos patients, with an average long-term survival rate around 50% (Table 4). Notably, the GRAALL study contributed a significant information in clinically HR patients aged 15–55 years (n=522), who were subject to MRD analysis although an allo-SCT was prescribed by design.^39^ In this study, 238 of 522 total patients had a transplant (54%). When outcome was considered by MRD status, available for 278 patients, it was found that an allo-SCT did not benefit the MRD-responsive group (molecular MRD <10^−4^ at week 6, superimposable relapse-free survival rates close to 70% at 4 years), whereas it improved outcome of MRD-pos group (P=0.04), the survival rate increasing from about 30% without allo-SCT to about 50% at 4 years. The basic question is, therefore, how to predict cure by allo-SCT in MRD-pos patients, in order to shift to new experimental therapies prior to and/or instead of an allo-SCT in patients unlikely to benefit from this procedure. The final long-term update of the NILG trial^34^ indicated how the risk of post-transplantation relapse in MRDpos patients correlated with post-induction quantitative MRD peaks at planned study time-points (weeks 10, 16 and 22), regardless of the time elapsed from CR to MRD analysis and/or subsequent SCT. Patients undergoing allo-SCT with one or more post-induction MRD reads of 10^−3^ and greater had an inferior outcome (5-year survival 20% vs. 60% with all post-induction MRD reads <10^−3^). The 10^−3^ MRD level could be critical for the decision to transplant or not MRDpos patients.

## The Future of MRD Analysis in Ph- ALL

Fifteen years of MRD-based clinical studies established the dominant prognostic role of MRD, confirmed the value of MRD-based clinical trials and allowed to identify the majority of the patients who are likely to be cured by chemotherapy with no or little treatment-related mortality. These latter become early and persistently MRDneg, with no relationship to their clinical risk class. Instead, the patients displaying either MRD persistence or relapse are not curable by chemotherapy and may be cured by SCT (despite the higher death risk), although in a lower proportion than unselected ALL patients, in relation with quantitative MRD ranges and other undefined factors. The predictive power of MRD analysis is not absolute because 20–30% of MRD responsive patients will relapse, and certainly needs to be improved. At the same time, the risk definition given by MRD is as yet unrivalled, representing the disease itself, serially measured at the submicroscopic level in response to the anti-leukemic therapy, which is an extremely useful information. If well used, this instrument offers the best chance to individualize and optimize treatments on a sound, rational basis. In the end, coupling modern PDT concepts for induction/consolidation therapy with an improved MRD-based and risk-based transplantation strategy focusing on early, clinically meaningful time-points, may open the way to real therapeutic progress. As an example, in the recent PDT/MRD-based NILG study (n=140; age range 18–60 years), week 10 MRD response was increased to 72% and 4-year survival rate was 64%.^40^ For poor MRD responders and all those unlikely to benefit from allo-SCT, cytotoxic monoclonal antibodies like inotuzumab ozogamicin and blinatumomab as well as chimeric antigen receptor-modified T cells represent new exciting therapeutic possibilities.^41^–^43^

## Figures and Tables

**Table 1:** Current risk definitions adopted by leading European groups for adult ALL (adapted and updated from: R Bassan et al., Prognostic factors and Risk-Adapted Therapy. In: *Recommendations of the European Working group for Adult ALL*. N Goekbuget et al (eds.), UNI-MED 2011,40–52. GIMEMA and NILG: Italy; GMALL: Germany; HOVON: The Netherlands; PALG: Poland; PETHEMA: Spain; UK NCRI: United Kingdom.

	GIMEMA	GMALL	GRAALL	HOVON	NILG	PALG	PETHEMA	UK NCRI
Age (years)	−	- (max 55)	- (max 60)	−	- (max 65)	>35	>30	>40
WBC (x10^9^/L)	>50	>30 (B)	>30 (B)	>30 (B), >100 (T)	>30 (B), >100 (T)	>30	>30	>30 (B), >100 (T)
Late CR	+	+	+	+	+	+	+	+
Cytogenetics	t(4;11), t(1;19)	t(4;11)	t(4;11), other adverse	t(4;11), other adverse	t(4;11), other adverse	t(4;11)	t(4;11)	t(4;11), other adverse
Phenotype	−	B-I, T-I/II/IV	CD10-neg	−	B-I, T-I/II/IV	B-I, T-I/II/IV	−	−
MRD	NE (→ +)	+	NE (→ +)	NE	+	+	+	+
Other	PPR	−	CNS-pos, PPR/d8R	−	−	−	−	−

WBC, white blood cells; PPR, poor prednisone response; d8R, day 8 chemo-resistance, NE, not evaluated

**Table 2:** Targets, advantages and disadvantages of different techniques for MRD detection.

Target	Technique	Applicability	Sensitivity	Advantages	Disadvantages
Ig/TCR gene rearrangements	RQ-PCR	Up to 90%	10^−4–^10^−5^	-High sensitivity -High standardization level -Large applicability	-Time-consuming -Target instability -Large experience needed
Fusion transcripts	RQ-PCR	30–40% (adult-pediatric)	10^−4–^10^−5^	-High sensitivity -Target stability during the disease course -Rapidity	-Instability of RNA -Quantification not linked to leukemia cell number -Cross-contamination risk -Applicable to a proportion of patients
Leukemia-Associated Immunophenotype	Multicolor Flow Cytometry	>90%	10^−3–^10^−4^	-Relatively high sensitivity -Large applicability -Rapidity	-Difficult to standardize -Large experience needed -Phenotype switch
Ig/TCR gene rearrangements	NGS	>90%	At least 10^−5^	-Highest reported sensitivity -Uniform analysis between diagnosis and follow-up -Large applicability	-Not yet standardized

**Table 3:** Results of prospective, MRD-based clinical trials in Ph- ALL (GMALL, NILG and PETHEMA also used MRD to orientate treatments). In GMALL trial, the high complete MRD response rate (molCR) may partly reflect the numerical predominance of SR patients (SR 434 and HR 146, see also footnote no. 4). In PETHEMA trial, the high MRD response rate may be in relation with the lower sensitivity threshold of MFC analysis. In GRAALL trial, the variable CIR rates reflect outcome of patients with negative MRD or MRD <10^−4^ at given time-point, respectively; the variable hazard ratios and P values reflect results of M/V analysis in patients with B- and T-ALL, respectively.

Study (year started)	Patient no. (risk class)	MRD method	MRD response definition	No. MRD responsive (%)	MRD-based therapy^1^	Outcome (vs MRD-unresponsive)^2^	M/V statistics^3^ (risk factors for relapse)
GMALL (1999)	580 (SR, HR)	RQ-PCR	MRD negative @ w10 and @ w16	407 (70)^4^	SR only	5-year CCR 74% vs 35% (P<0.0001); OS 80% vs 42% (P<0.0001)	MRD positive (HR 4.5; P<0.0001)
NILG (2000)	136 (SR, HR)	RQ- PCR	MRD <10^−4^ @ w16 and negative @ w22	76 (56)	SR and HR t(4;11)-	6-year DFS 66% vs 25% (P=0.000); OS 75% vs 32% (P=0.000)	MRD positive (HR 5.3; P=0.001); WBC >100 (HR 2.2; P=0.005)
PETHEMA (2003)	161 (HR)	MFC	MRD <5 x10^−4^ @ w18^5^	139 (86)	HR	5-year DFS 55% vs 32% (P=0.002); OS 59% vs 37% (P=0.002)	MRD positive (HR 3.7; P<0.001)
GRAALL (2003)	423 (SR, HR)	RQ- PCR	MRD <10^−4^ after induction @ w6	265 (63)	N/A^6^	5-year CIR 23–31% vs 60% (P=0.002)	MRD positive (HR 2.49–4-39; P=0.001–0.002); oncogenetics (HR 1.75–4.39; P=0.05- 0.002)^7^

1 chemotherapy if MRD-responsive; allo-SCT if MRD-unresponsive,

2 by treatment intention; CCR, continuous CR; OS, overall survival; DFS, disease-free survival; CIR, cumulative incidence of relapse,

3 M/V, multivariable,

4 molCR: SR 77%, HR 51% (P<0.0001),

5 including day 14 blast cell clearance in MRD responsive group,

6 not applicable (MRD results not used to orientate treatment),

7 see text for details.

**Table 4:** Results of allogeneic SCT performed in CR1 in MRD+ patients with Ph- ALL (data from prospective MRD-oriented trials of the GMALL, NILG and PETHEMA Groups).

Study (year started)	MRD+ (no.)	MRD+ to allo-SCT, no. (%)	Outcome allo-SCT^1^	Outcome no allo-SCT^1^	P value
GMALL (1999)	120	57 (47)	5-year DFS 44% 5-year OS 54%	DFS 11% OS 33%	<0.001 0.06
NILG (2000)	60	26 (43)	6-year DFS 42%	DFS 12%	0.000
PETHEMA (2003)	24	24	5-year DFS 24% 5-year OS 31%	-	-
